# Respiratory Syncytial Virus: Willingness towards a Future Vaccine among Pregnant Women in Italy

**DOI:** 10.3390/vaccines11111691

**Published:** 2023-11-04

**Authors:** Grazia Miraglia del Giudice, Vincenza Sansone, Francesca Airoma, Silvia Angelillo, Francesca Licata, Gabriella Di Giuseppe

**Affiliations:** 1 Department of Experimental Medicine, University of Campania “Luigi Vanvitelli”, 80138 Naples, Italy; grazia.miragliadelgiudice@unicampania.it (G.M.d.G.); vincenza.sansone@unicampania.it (V.S.); francesca.airoma@studenti.unicampania.it (F.A.); 2 Department of Health Sciences, University of Catanzaro “Magna Græcia”, 88100 Catanzaro, Italy; silvia.angelillo@studenti.unicz.it (S.A.); f.licata@unicz.it (F.L.)

**Keywords:** cross-sectional study, Italy, pregnancy, RSV, vaccine, willingness

## Abstract

Background: This cross-sectional survey was designed to evaluate pregnant women’s awareness regarding Respiratory Syncytial Virus (RSV) infection and willingness to receive the vaccine during pregnancy and to vaccinate their newborn against RSV. Methods: An anonymous survey was administered from 20 April to 30 June 2023, to pregnant women aged ≥ 18 years attending gynecology wards of randomly selected public hospitals in southern Italy. A minimum sample size of 427 participants was calculated. The survey assessed women’s socio-demographic characteristics, health-related information, their source(s) of information, and attitudes regarding RSV. Results: A total of 490 women participated. Those who were married/cohabiting, with a high-school degree compared to those who had a university degree, and those who needed additional information were more concerned that the newborn could acquire the RSV infection. The perceived utility of a future RSV vaccine administered during pregnancy was higher among those who were married/cohabiting, with a university degree, those with very good perceived health status, those who received information from healthcare workers, and those who needed additional information. Only 45.9% were willing to be vaccinated during pregnancy, and this was more likely among those with a university degree, with a very good perceived health status, who had received information from healthcare workers, and who needed more information. Finally, almost two-thirds (61.1%) were willing to vaccinate their newborn, and this was more likely among women with a university degree, with a very good perceived health status, and who needed additional information. Conclusions: An education campaign regarding RSV infection and its vaccine is needed in order to improve women’s perception and to support healthcare workers in promoting it when it will be available.

## 1. Introduction

Bronchiolitis is the most frequent lower respiratory tract infection and the leading cause of hospitalization and potentially of death in infants, with high health costs [1,2]. Along with various etiological agents responsible for bronchiolitis (influenza, adeno, and rhinovirus), Respiratory Syncytial Virus (RSV) is a common respiratory pathogen, especially in children under two years of age [3]. It has been estimated that RSV infects over 60% of children during the first year of life and almost all children by the second year of life, it being the second cause of death in infants [4]. RSV is responsible for seasonal infection, with annual epidemic peaks during the winter months [5]. In healthy people, RSV infection results in a flu-like syndrome, usually with mild symptoms. However, the most vulnerable individuals, such as infants, premature infants, and immunocompromised children, have a high risk of contracting a severe form of bronchiolitis or of developing complications [6,7,8]. Globally, RSV infection affects approximately 33 million children under the age of five, with 3.2 million hospitalizations and 120,000 deaths each year [9,10]. In Europe, an average of 245,244 (95% confidence interval [CI], 224,688–265,799) hospital admissions with a respiratory infection per year were associated with RSV in children under the age of five, especially among children under the age of one year (75%) [11]. In recent years, moreover, an increase in the diagnosis of bronchiolitis and the consequent admissions to pediatric intensive care units (PICU) among children has been observed [12,13]. Indeed, during the COVID-19 pandemic, the preventive measures adopted, such as lockdown, travel restrictions, and physical distancing, that were introduced to reduce the spread of the SARS-CoV-2 virus, also reduced the circulation of RSV [14]. With the lowering of the preventive measures, RSV infection has seen peaks in atypical periods and a considerable impact on healthcare systems worldwide [15,16,17,18]. In Italy, from the beginning of the last winter season until week 05/2023, for a total of 17,568 clinical samples collected in different laboratories, 27% tested positive for influenza virus, 15.8% for RSV, 7.1% for SARS-CoV-2, and 6.9% for other respiratory viruses [19].

To date, there is no specific therapy for RSV; therefore, treatment is oriented only toward symptom control. In Italy, the administration of a monoclonal antibody—palivizumab—for the prevention of infections caused by RSV is indicated only in high-risk children, such as children born with a gestational age ≤ 35 weeks and less than 6 months of age at the onset of the seasonal RSV epidemic [20].

Therefore, one target population for vaccination is pregnant women, which would protect the newborn through transplacental transfer of maternal antibodies or breastfeeding [21]. The Food and Drug Administration (FDA) has approved the first RSV vaccine in the United States for the prevention of respiratory disease caused by RSV in individuals 60 years of age and older [22]. Moreover, in July, the European Medicines Agency (EMA) recommended granting a marketing authorization in the European Union for a vaccine to protect against disease caused by RSV in adults aged 60 years and older and in infants from birth through 6 months of age with passive immunization following administration of the vaccine to the mother during pregnancy [23]. With this premise, this cross-sectional survey was designed to evaluate pregnant women’s awareness regarding RSV infection and willingness to adhere to vaccinating against RSV during pregnancy and to vaccinate their newborn when a vaccine is available.

## 2. Materials and Methods

### 2.1. Setting and Study Population

The present cross-sectional survey was undertaken from 20 April to 30 June 2023 and involved pregnant women or those who had just given birth (in the 2 days before the interview), aged ≥ 18 years, and attending a gynecology ward. A two-stage sampling strategy was designed. Public hospitals with gynecology wards were randomly selected from the geographic areas of Naples and Catanzaro, Italy. The sample size was calculated using a single population proportion formula, with the assumption that 50% of pregnant women were willing to receive the vaccination against RSV during the pregnancy, using a confidence level of 95%, a margin of error of 5%, and considering a response rate of 90% [24]. Therefore, at least 475 pregnant women had to be invited in order to achieve a final sample size of 427 pregnant women. Before starting data collection, the project was presented to the director of public hospitals to obtain permission and collaboration. Once approval to join the project was obtained, every potential participant was approached by experienced trained personnel while waiting for their appointment with their gynecologist/obstetrician or while they were hospitalized in the gynecology ward. Pregnant women received a letter and an informed consent form (Supplementary Material). The letter briefly described the study’s aims. Women were assured that joining the project was voluntary and that confidentiality of responses would be granted, and they were invited to sign the consent form. After obtaining written informed consent, the research team conducted a face-to-face interview. The participants did not receive any incentives or gifts. 

The study protocol was approved by the Ethics Committee of the Teaching Hospital of the University of Campania “Luigi Vanvitelli” (0016978/I 7 2023).

### 2.2. Survey Instrument

The time needed to answer the survey was approximately 10 min, and it was structured into three sections (Supplementary Material). The first section was designed to collect women’s socio-demographic characteristics and health-related information, including age, marital status, level of education, employment status, number of children, underlying chronic medical conditions, and self-perceived health status during pregnancy, measured on a 10-point Likert scale, where “one” was “very bad” and “ten” was “very good”. The second section contained three questions to assess whether they had ever heard about RSV, the source(s) from which they gathered information, and if the respondents perceived the need for additional information. Participants who had never heard about RSV infection were provided with a summary of it in order to balance knowledge before proceeding with the questionnaire. The third section consisted of 5 questions regarding the attitudes about RSV and its vaccine. Three questions, regarding the concern that the newborn could acquire the RSV infection, the perceived utility of a future vaccine against RSV administered during pregnancy, and the perceived utility to vaccinate their newborn against RSV when a vaccine is available were collected on a 10-point Likert scale, where “one” was “not at all” and “ten” was “very much”. The other two questions were in regards to the willingness to receive a vaccine against RSV during pregnancy and to immunize their newborn against this infection, with “yes”, “no”, and “do not know” responses. The reason(s) regarding their willingness or unwillingness were explored by using a close-ended multiple-choice question, in which respondents could select as many reasons as appropriate for their decision. A pilot study among 50 pregnant women was carried out to evaluate the readability, clarity, and correct flow of the items. As a result of the pilot study, no questions were amended. Therefore, the data collected in the pilot study were included in the final survey.

### 2.3. Statistical Analysis

All analyses were performed with Stata software version 18 [25]. First, descriptive statistics, including relative frequency for categorical variables and mean and standard deviation for continuous factors, were used to summarize the main characteristics of the respondents. Second, univariate analyses were performed using the chi-square test, the Student’s *t*-test, and the analysis of variance (ANOVA) to evaluate the association between several potential determinants and (1) concern that the newborn could acquire the RSV infection (continuous), (2) perceived utility of a future vaccine against RSV administered during pregnancy (continuous), (3) willingness to receive a future vaccine against RSV during pregnancy (no/do not know = 0; yes = 1), and (4) willingness to immunize their newborn with a future vaccine against RSV (no/do not know = 0; yes = 1). In the multivariate regression models, the following independent variables, which were judged to potentially influence the above-mentioned outcomes (Models 1–4), were included: age group, in years (≤32 = 0; >32 = 1), marital status (unmarried/separated/divorced/widowed = 0; married/cohabiting = 1), level of education (no formal education/primary school/secondary school = 0; high school = 1; university degree = 2), employed (no = 0; yes = 1), number of other children (0 = 0; 1 = 1; >1 = 2), third trimester of pregnancy (no = 0; yes = 1), self-perceived health status during pregnancy (1–9 = 0; 10 = 1), having received information about RSV from healthcare workers (no = 0; yes = 1), and need of additional information (no = 0; yes = 1). The following variables were also included in Models 2 and 3: underlying chronic medical condition (no = 0; yes = 1) and pregnancy at risk (no = 0; yes = 1). Results are presented as odds ratios (ORs) and 95% confidence intervals (CIs). All reported *p*-values are two-tailed, and a value ≤ 0.05 was considered statistically significant.

## 3. Results

### 3.1. Socio-Demographic and Anamnestic Characteristics of the Respondents

Of the 533 pregnant women invited, 490 agreed to participate in the study for a response rate of 92%, and the main mothers’ characteristics are described in Table 1. A large majority of responding women (87.2%) were in the third trimester of pregnancy, the average age was 31.9 years, most were married or cohabiting (90.4%), more than a third had a university degree (36.5%), more than two-thirds were employed (63.3%), more than a third (38.1%) were at the first pregnancy, and 26% had more than one child. Risk factors in pregnancy were reported in 18.6% of the respondents, the majority (68.1%) of them had diseases related to pregnancy, and 14.5% had chronic medical condition. When pregnant women were asked about their perceived health status in pregnancy, 16.2% reported a very good health status with an overall mean value of 7.8 out of a maximum score of 10.

### 3.2. Pregnant Women’s Attitudes Regarding RSV Infection and Related Vaccination

When pregnant women were asked about their attitudes, 20% of them were very concerned about their children’s risk of contracting RSV infection, with an overall mean value of 6.7 out of a maximum score of 10. As shown in Table 1, univariate analyses have evidenced the significant associations of several variables with the outcomes of interest, which have been partially confirmed by multivariate analyses (Table 2). The stepwise linear regression analysis, performed to estimate predictors of the concern that the newborn could acquire the RSV infection, showed that pregnant women married or cohabiting, those with a high-school degree compared to those who had a university degree, and who needed additional information were significantly more concerned (Table 2, Model 1). Moreover, the RSV vaccine administered during pregnancy was considered very useful for protecting newborns only by 15.5% of participants, with an overall mean value of 6.4 out of a maximum score of 10. The stepwise linear regression analysis showed that the vaccine is considered more useful by women married or cohabiting, by those with a university degree compared to those with no formal education/primary-school/secondary-school degree, those with a very good perceived health status during pregnancy, those who received information about RSV from healthcare workers, and participants who needed additional information (Table 2, Model 2). Finally, the RSV vaccine for newborns was perceived to be very useful for 21.7% of pregnant women, with an overall mean value of 7 out of a maximum score of 10.

Investigating the willingness of pregnant women to receive a future vaccine against RSV during pregnancy showed that 45.9% were willing to vaccinate themselves. The stepwise logistic regression analysis showed that women with a university degree compared with those who had a high-school degree and those with no formal education/primary-school/secondary-school degree, those with a very good perceived health status during pregnancy compared with those who did not have a very good perceived status during pregnancy, those who received information about RSV from healthcare workers compared with those who did not receive information from healthcare workers, and those who needed additional information compared with those who did not need additional information were more likely to be willing to vaccinate themselves during pregnancy (Table 2, Model 3). The reported reasons for this were the need to protect the newborn (70.5%), concerns over the safety of the vaccine (35.3%), having a recommendation from the gynecologist (30.5%), effectiveness of the vaccine (24.5%), trust in the effectiveness of the vaccines (20.5%), and concerns regarding the severity of RSV infection (12.5%). The reasons for refusing to vaccinate themselves during pregnancy against RSV included concern regarding side effects (50.6%), the safety of the vaccine (20%), the uselessness of the vaccine (9.4%), the ineffectiveness of the vaccine (7.5%), a lack of trust in vaccinations (10.2%), and the belief that RSV infection does not cause a severe disease (2.6%). Overall, 61.1% declared their willingness to vaccinate their newborn with a future vaccine against RSV. The stepwise logistic regression analysis showed that women with a university degree compared with those with a high-school degree and those with no formal education/primary-school/secondary-school degree, those with a very good perceived health status during pregnancy compared with those who did not perceive a very good health status, and pregnant women who needed additional information compared with those who did not need additional information were more likely to be willing to vaccinate their newborn (Table 2, Model 4). The reported reasons were to protect the newborn (75.6%), having received a recommendation from the primary care pediatrician (32.4%), safety (31.8%) and effectiveness (20.4%) of the vaccine, general trust in the effectiveness of the vaccines (15.4%), and concerns regarding the severity of RSV infection (11%). The reasons for refusing to vaccinate their newborn with a future vaccine against RSV included concerns regarding side effects (41.6%), a belief that the vaccine was unsafe (14.2%), lack of trust in vaccinations (11.4%), uselessness of the vaccine (10.5%), ineffectiveness of the vaccine (9.5%), and the belief that RSV infection does not cause a severe disease (4.2%).

### 3.3. Sources of Information

Only a third of pregnant women (32.2%) reported to have heard about the RSV infection, and most of them received information from healthcare workers (39.9%), followed by family members or friends (28.5%) and media (23.4%). Moreover, more than two-thirds of the respondents (64.7%) reported the need to receive additional information about a future vaccine against RSV.

## 4. Discussion

As is well known, vaccines have transformed public health and vaccinations are the most effective interventions for the primary prevention of infectious diseases. In addition, it is important to underline that receiving a vaccination during pregnancy allows women to produce antibodies that will protect their newborn through transplacental transfer and breastfeeding. As far as we know, the present study is the first survey conducted in Italy and designed to collect detailed data on the willingness of pregnant women to receive a future vaccine against RSV during pregnancy and to vaccinate their newborn. Therefore, the results of this study would add knowledge to this topic and may be useful for offering valuable support to decision makers when designing strategies to promote future RSV vaccination among this population.

The main findings of this survey indicate that only 20% and 15.5% of the participants were very concerned about their children’s risk of contracting RSV infection and considered the RSV vaccine administered during pregnancy very useful for protecting newborns, respectively. Moreover, less than half of respondents (45.9%) were willing to vaccinate themselves during pregnancy, whereas almost two-thirds (61.1%) declared their willingness to vaccinate their newborn with a future vaccine against RSV.

The first key finding is that most of the sample (67.8%) had never heard about RSV infection. However, an even lower level of awareness has been previously reported in England and in Australia, with only 29% and 17%, who knew about RSV, respectively [26,27]. This result is upsetting since RSV-associated lower respiratory tract infection represents one of the most common causes of death among infants [4]. Therefore, the crucial importance of spreading information in this population is evident. Moreover, given this finding, it is not surprising that pregnant women in this survey reported a low level of concern that their children could get RSV infection and had an even lower perception of the utility of a future vaccine against RSV administered during pregnancy. 

The second key finding was that only 45.9% reported their willingness to receive a future RSV vaccine during pregnancy. A significantly higher willingness has been observed in England, where 75% would accept RSV vaccines if routinely recommended [27]. It is worth mentioning that low levels of willingness among pregnant women have also been confirmed for other recommended vaccinations: only 3.6%, 29.7%, and 41% were willing to receive the COVID-19 vaccine during pregnancy in, respectively, Czechia, Switzerland, and the USA [28,29,30], whereas in Tunisia 36.8% were willing to accept the influenza vaccine [31], and a study conducted in Italy showed that only 27.9% of pregnant women were willing to receive all the recommended vaccines during pregnancy [32]. However, the proportion presented in this survey was significantly lower than what has been observed in other studies conducted among pregnant women regarding other vaccines: 76.3% for the influenza vaccination in China [33], 76.3% for the Tdap (tetanus, diphtheria, pertussis) vaccine in Canada [34], and 72.8% and 71.9% for the COVID-19 vaccine in Canada and in Italy, respectively [24,35]. Such differences can be justified by the different periods of time in which the studies were conducted, the methodology used, and the characteristics of the samples.

It is interesting to note that the main reasons mentioned by the participants for the unwillingness to receive the RSV vaccine during pregnancy were concerns regarding side effects (50.6%) and a belief that the vaccine was unsafe (20%). It has been broadly proven that concerns about vaccine safety can be a barrier to willingness to accept a vaccination [24,28,34,36,37,38], since the safety of a new vaccination is considered more important than its efficacy [26]. Women can show hesitancy toward vaccines only during pregnancy due to the tendency to link the potential adverse effects of vaccines to the fetus and to the inadequate risk perception of severe infection among unborn children [38]. Therefore, future intervention promoting RSV vaccination during pregnancy should address the concerns of expectant mothers, focusing on the safety data of the vaccine. In addition, education should shed light on the risk of severe illness that infants may experience and on the possibility of prevention by a vaccine.

However, the most common reasons for willingness to receive the RSV vaccine were to protect the newborn (70.5%) and a belief that the vaccine is safe (35.3%). It should be underlined that a positive attitude towards vaccine safety has been widely confirmed as a key determinant for increasing vaccination uptake [32,39,40,41].

These findings can be of great help for policy makers to address and identify obstacles to the acceptance of the RSV vaccine.

The third notable finding to mention is that a high number of respondents (61.1%) was willing to vaccinate their newborn with a future vaccine against RSV. This could be explained by assuming that vaccinations among children are more common and accepted by the population, whereas vaccinations administered during pregnancy still lack trust. 

Furthermore, the multivariate linear and logistic regression analysis have found associations between several independent variables and the different outcomes of interest. Among the socio-demographic characteristics, the level of education was associated with all our outcomes of interest. Indeed, those with a university degree were more likely to be less concerned about their child’s risk of contracting RSV infection and to have a higher perception of the utility of the vaccine, as well as to be willing to both receive the vaccine during pregnancy and to vaccinate the child. This result is particularly interesting and underlines how a higher level of education has a positive impact on attitudes and willingness towards vaccines, as already demonstrated in the literature [24,29,30,32,36,42,43], suggesting that particular care should be given in clinical practice to low-educated populations.

Moreover, married women were more likely to consider the RSV vaccine useful. Not being married has already been described as a barrier for vaccination in previous studies; therefore, this result suggests that interventions should be targeted at this population [44,45]. It is also notable to mention that a very good perceived health status during pregnancy was among the key drivers for willingness to receive the vaccine, probably because women with a worse health status are more likely to be worried that the vaccination could exacerbate their health conditions [44,46].

Finally, women who received information about RSV from HCWs were more likely to believe that the vaccine was useful as well as to be willing to receive the vaccine during pregnancy. Nowadays, it is confirmed by a vast amount of literature that HCWs play a key role in positively influencing attitudes regarding vaccinations, and consequently their uptake [34,35,36,47]. Therefore, these results highlight the need to advise HCWs when recommendations regarding the RSV vaccine during pregnancy will be available in order to contrast misinformation and help women to make a decision. Keeping HCWs aware of the most recent evidence-based guidelines regarding vaccinations has been proposed as a key factor in informing and recommending vaccination during pregnancy [48,49].

The results of this survey are particularly interesting since infants in the first 2 months of life have a higher risk of developing a severe form of RSV infection and a vaccine administered during pregnancy could give protection during this period of elevated risk [50]. Therefore, a maternal RSV vaccine may have very significant health benefits, reducing infant morbidity and mortality, as already shown by the near elimination of neonatal tetanus thanks to maternal vaccination [51]. However, a major concern about a future RSV vaccine administered during pregnancy is related to the limited transplacental antibody transfer that occurs among children born prematurely, who are also those at higher risk of a severe RSV infection. For this reason, a combined approach with both maternal vaccination and consequent infant immunization may also be possible to guarantee better protection. 

The intrinsic limitations of cross-sectional studies should be taken into consideration when interpreting the findings. First, there is the inability to determine temporal sequentiality and to assess casual relationships between variables. Second, all the information was self-reported and a social desirability bias might be possible; in addition, the women were not asked if they were antivaxxers. However, anonymity was guaranteed; therefore, the findings are likely to be reliable. Despite these limitations, a fundamental strength of this survey is that it is the first conducted in Italy reporting the willingness to accept a future vaccine against RSV among pregnant women.

## 5. Conclusions

In conclusion, this survey clearly indicates a lack of awareness among pregnant women about RSV infection. An education campaign regarding a future RSV vaccine and knowledge regarding its safety and efficacy are needed in order to improve women’s perception and to support HCWs in promoting this future vaccine.

## Figures and Tables

**Table 1:** Results of univariate analysis exploring the characteristics associated with the outcomes of interest in the study population.

Characteristics	Total (N = 490)		Concern that the Newborn Could Acquire the RSV Infection (N = 490)	Perceived Utility of a Future Vaccine against RSV Administered during Pregnancy (N = 489)	Willingness to Receive a Future Vaccine against RSV during Pregnancy (N = 489)		Willingness to Immunize Their Newborn with a Future Vaccine against RSV (N = 489)	
	n	%	Mean ± SD	Mean ± SD	n	%	n	%
Age group (years) (489) ^a^	31.9 ± 4.8 (18–47) ^b^							
≤32	256	52.3	6.6 ± 2.9	6.3 ± 2.7	104	40.8	152	59.6
>32	233	47.7	6.7 ± 2.6	6.6 ± 2.8	120	51.5	147	63.1
			*t*-test (489) = −0.71, *p* = 0.474	*t*-test (488) = −0.97, *p* = 0.331	χ^2^ = 5.63, df = 1, *p* = 0.018		χ^2^ = 0.62, df = 1, *p* = 0.43	
Marital status (489) ^a^								
Unmarried/separated/ divorced/widowed	47	9.6	5.6 ± 2.8	5.5 ± 2.9	24	51.1	30	63.8
Married/cohabiting	442	90.4	6.8 ± 2.7	6.5 ± 2.7	200	45.3	269	61
			*t*-test (489) = −2.71, *p* = 0.006	*t*-test (488) = −2.35, *p* = 0.019	χ^2^ = 0.55, df = 1, *p* = 0.455		χ^2^ = 0.14, df = 1, *p* = 0.705	
Level of education								
No formal education/elementary school/secondary school	87	17.8	6.3 ± 3.2	5.7 ± 3	39	33.3	42	48.3
High school	224	45.7	6.8 ± 2.6	6.2 ± 2.6	90	40.4	124	55.6
University degree	179	36.5	6.6 ± 2.6	7.1 ± 2.5	105	58.7	133	74.3
			ANOVA-test (490) = 18.45, *p* = 0.291	ANOVA-test (488) = 137.49, *p* ≤ 0.001	χ^2^ = 20.03, df = 2, *p* < 0.001		χ^2^ = 21.98, df = 2, *p* < 0.001	
Employed								
No	180	36.7	6.6 ± 3	6.1 ± 3	69	38.3	94	52.2
Yes	310	63.3	6.7 ± 2.5	6.6 ± 2.6	155	50.2	205	66.3
			*t*-test (490) = −0.13, *p* = 0.897	*t*-test (489) = −2.09, *p* = 0.036	χ^2^ = 6.41, df = 1, *p* = 0.011		χ^2^ = 9.54, df = 1, *p* = 0.002	
Number of children (488) ^a^								
0	186	38.1	6.8 ± 2.7	6.5 ± 2.7	83	44.6	108	58.1
1	175	35.9	6.6 ± 2.8	6.5 ± 2.6	79	45.4	113	64.9
>1	127	26	6.4 ± 2.8	6.1 ± 3	60	47.2	76	59.8
			ANOVA-test (488) = 14.98, *p* = 0.366	ANOVA-test (486) = 21.11, *p* = 0.241	χ^2^ = 0.21, df = 2, *p* = 0.899		χ^2^ = 1.88, df = 2, *p* = 0.39	
Third trimester of pregnancy (484) ^a^								
No	62	12.8	7.1 ± 2.3	6.4 ± 2.3	32	51.6	42	67.7
Yes	422	87.2	6.6 ± 2.8	6.4 ± 2.8	192	45.6	256	60.8
			*t*-test (484) = 1.26, *p* = 0.209	*t*-test (483) = 0.06, *p* = 0.953	χ^2^ = 0.78, df = 1, *p* = 0.376		χ^2^ = 1.09, df = 1, *p* = 0.294	
Pregnancy at risk (489) ^a^								
No	398	81.4	-	6.4 ± 2.7	178	44.8	-	-
Yes	91	18.6	-	6.3 ± 2.9	46	50.5	-	-
			-	*t*-test (488) = 0.44, *p* = 0.659	χ^2^ = 0.97, df = 1, *p* = 0.324		-	
Underlying chronic medical condition (488) ^a^								
No	417	85.5	-	6.4 ± 2.7	186	44.7	-	-
Yes	71	14.5	-	6.6 ± 2.7	36	50.7	-	-
			-	*t*-test (487) = −0.45, *p* = 0.648	χ^2^ = 0.88, df = 1, *p* = 0.349		*-*	
Very good self-perceived health status during pregnancy (483) ^a^								
No	405	83.8	6.6 ± 2.7	6.2 ± 2.7	175	43.3	239	59.2
Yes	78	16.2	6.9 ± 2.9	7.4 ± 2.5	45	57.7	56	71.8
			*t*-test (483) = −0.81, *p* = 0.421	*t*-test (483) = −3.51, *p* < 0.001	χ^2^ = 5.44, df = 1, *p* = 0.02		χ^2^ = 4.39, df = 1, *p* = 0.036	
Having received information about RSV from healthcare workers								
No	427	87.1	6.6 ± 2.8	6.3 ± 2.8	182	42.6	255	59.7
Yes	63	12.9	7.1 ± 2.2	7.6 ± 2.1	42	67.7	44	71
			*t*-test (490) = −1.34, *p* = 0.181	*t*-test (489) = −3.59, *p* < 0.001	χ^2^ = 13.76, df = 1, *p* < 0.001		χ^2^ = 2.88, df = 1, *p* = 0.089	
Need of additional information (465) ^a^								
No	164	35.3	5.9 ± 2.9	5.6 ± 2.9	60	36.6	84	51.2
Yes	301	64.7	7.1 ± 2.5	6.9 ± 2.5	159	52.8	202	67.1
			*t*-test (465) = −5.12, *p* < 0.001	*t*-test (465) = −4.97, *p* < 0.001	χ^2^ = 11.23, df = 1, *p* = 0.001		χ^2^ = 11.32, df = 1, *p* = 0.001	

^a^ Number of each item may not add up to total number of study population due to missing values. In parenthesis is the number of respondents to each item. ^b^ Mean ± Standard deviation (range). Respiratory Syncytial Virus (RSV).

**Table 2:** Linear and logistic regression models.

Variable	Coeff	SE ^a^	T	*p*
Model 1. Concern that the newborn could acquire the RSV infection F (9, 442) = 4.53; R^2^ = 8.4%; adjusted R^2^ = 6.6%; *p* < 0.0001				
Need of additional information (No = 0; Yes = 1)	1.26	0.27	4.69	<0.001
Married/cohabiting (No = 0; Yes = 1)	1.23	0.43	2.82	0.005
Level of education				
High school	0.71	0.29	2.43	0.015
No formal education/primary school/secondary school	0.55	0.39	1.41	0.158
University degree	Reference category			
Having received information about RSV from healthcare workers (No = 0; Yes = 1)	0.72	0.38	1.91	0.057
Number of children				
>1	−0.43	0.29	−1.45	0.147
0	1.00 *			
Age group (years)				
≤32	Reference category			
>32	0.32	0.26	1.2	0.232
Third trimester of pregnancy (No = 0; Yes = 1)	−0.44	0.38	−1.15	0.251
Perceived health status during pregnancy (1–9 = 0; 10 = 1)	0.32	0.33	0.97	0.33
Model 2. Perceived utility of a future vaccine against RSV administered during pregnancy F (6, 443) = 9.87; R^2^ = 11.8%; adjusted R^2^ = 10.6%; *p* < 0.0001				
Perceived health status during pregnancy (1–9 = 0; 10 = 1)	1.19	0.33	3.63	<0.001
Need of additional information (No = 0; Yes = 1)	1.02	0.26	3.85	<0.001
Having received information about RSV from healthcare workers (No = 0; Yes = 1)	1.17	0.37	3.16	0.002
Level of education				
No formal education/primary school/secondary school	−0.87	0.37	−2.36	0.019
High school	−0.54	0.28	−1.94	0.053
University degree	Reference category			
Married/cohabiting (No = 0; Yes = 1)	0.98	0.43	2.31	0.021
Variable	OR ^b^	95% CI ^c^		*p*
Model 3. Willingness to receive a future vaccine against RSV during pregnancy Log likelihood = −288.63, χ^2^ = 45.07 (8 df), *p* < 0.0001 (sample size 450)				
Having received information about RSV from healthcare workers (No = 0; Yes = 1)	2.54	1.37–4.69		0.003
Perceived health status during pregnancy (1–9 = 0; 10 = 1)	1.94	1.14–3.31		0.015
Need of additional information (No = 0; Yes = 1)	1.68	1.12–2.56		0.015
Level of education				
No formal education/primary school/secondary school	0.48	0.25–0.92		0.027
High school	0.62	0.39–0.99		0.048
University degree	Reference category			
Having an underlying chronic medical condition (No = 0; Yes = 1)	1.4	0.81–2.43		0.229
Employed (No = 0; Yes = 1)	1.29	0.83–2.01		0.256
Age group (years)				
≤32	Reference category			
>32	1.22	0.82–1.83		0.324
Model 4. Willingness to immunize their newborn with a future vaccine against RSV Log likelihood = −280.96, χ^2^ = 38.62 (7 df), *p* < 0.0001 (sample size 452)				
Level of education				
No formal education/primary school/secondary school	0.42	0.22–0.79		0.007
High school	0.52	0.32–0.84		0.008
University degree	Reference category			
Need of additional information (No = 0; Yes = 1)	1.76	1.16–2.68		0.008
Perceived health status during pregnancy (1–9 = 0; 10 = 1)	1.98	1.12–3.5		0.019
Employed (No = 0; Yes = 1)	1.49	0.96–2.33		0.076
Number of children				
>1	1.51	0.9–2.52		0.116
1	1.42	0.89–2.28		0.136
0	Reference category			

^a^ Standard error. ^b^ Odds ratio. ^c^ Confidence Interval. * Reference category.

## Data Availability

The data presented in this study are available upon request from the corresponding author.

## Supplementary Materials

The following supporting information can be downloaded at: https://www.mdpi.com/article/10.3390/vaccines11111691/s1, Questionnaire: letter and informed consent form.
