# Supplementary material for: Respiratory Syncytial Virus: Willingness towards a Future Vaccine among Pregnant Women in Italy
Source: Vaccines (Basel). 2023 Nov 4;11(11):1691. doi: 10.3390/vaccines11111691 (PMC10674197; doi:10.3390/vaccines11111691)
Supplement: Supplementary file 1 [file vaccines-11-01691-s001.zip › vaccines-2665833-supplementary.pdf]

A. SOCIO-DEMOGRAPHIC AND ANAMNESTIC CHARACTERISTICS

This section is designed to gather information about your socio-demographic and anamnestic characteristics

A1. How old were you on your last birthday?________

A2. What is your marital status? □ Married □ Cohabiting □ Single □ Other *(please specifiy)*____________

A3. What is your highest educational level? □ None □ Primary school □ High school □ Baccalaurate □ Graduate

A4. What is your occupation?________________

A5. How many children do you have? (*please specify the number*) ______

A6. How many weeks are you pregnant? (*please specify*) ______

A7. Has your gynecologist considered your pregnancy at high risk? □ No □ Yes (*please specifiy the reason)*________________________

A8. Do you have any chronic medical condition? □ No □ Yes (*please specifiy, more than one disease is allowed*)________________

A9. On a scale from 1 to 10, how would you rate your health during pregnancy?

Not good 1 2 3 4 5 6 7 8 9 10 **Very good**

B. INFORMATION

This section is designed to explore your sources of information about the Respiratory Syncytial Virus (RSV) infection

B1. Have you ever heard about RSV infection? □No □Yes, from which sources?

□Healthcare workers *(please specify)* _________________ □Internet □ Mass media □Institutional organizations

□ Social media (Facebook, Twitter, Instagram, YouTube, etc.) □Friends and Family □Scientific journals

□Other *(please specify)*____

B2. Do you need further information about RSV? □No □Yes

Respiratory Syncytial Virus (RSV) is a very contagious virus that can infect the respiratory system of people of all ages, especially the elderly and children under the age of 2. Symptoms are generally similar to the common cold, however, it is possible to become seriously ill and develop pneumonia or bronchiolitis (inflammation of the small airways of the lungs). There are no specific drugs to treat RSV infection. In premature infants, especially those with cardiovascular diseases, lung problems, or immunological diseases, monoclonal antibodies can be used as a preventive measure. To date, there is no vaccination, but several studies are underway to develop one.

C. ATTITUDES

This section is designed to explore your attitudes toward RSV infection and its vaccination

C1. On a scale from 1 to 10, how concerned are you that your newborn could get the RSV infection? *(1 indicates that you are not concerned at all, 10 if you are very much concerned)*

Not at risk 1 2 3 4 5 6 7 8 9 10 **Very much at risk**

C2. On a scale from 1 to 10, how useful do you consider administering a future vaccination against RSV during pregnancy? (*1 indicates that you do not consider it useful at all, 10 if you consider it very useful)*

Not useful 1 2 3 4 5 6 7 8 9 10 **Very useful**

C3. On a scale from 1 to 10, how useful do you consider vaccinating your newborn with a future vaccination against RSV? (*1 indicates that you do not consider it useful at all, 10 if you consider it very useful)*

Not useful 1 2 3 4 5 6 7 8 9 10 **Very useful**

C4. If vaccination against RSV was available, would you vaccinate yourself during pregnancy?

□Yes | □No □Uncertain

Why would you vaccinate yourself? *(more than one answer is allowed)*	Why would you not vaccinate yourself? *(more than one answer is allowed)*
□ To protect the newborn from getting the infection	□ RSV vaccination does not protect the newborn from getting the infection
□ RSV infection can cause serious disease for my newborn	□ RSV infection does not cause serious disease for my newborn
□ If the gynecologist would recommend it	□ I am concerned about the side effects of RSV vaccination
□ If RSV vaccination would be safe	□ If RSV vaccination would not be safe
□ If RSV vaccination would be effective	□ If RSV vaccination would be effective
□ I trust in vaccines	□ I do not trust in vaccines

C5. If vaccination against RSV was available, would you vaccinate your newborn?

□Yes

□No □Uncertain

Why would you vaccinate yourself?<br>*(more than one answer is allowed)*	Why would you not vaccinate yourself?<br>*(more than one answer is allowed)*
□ To protect the newborn from getting the infection	□ RSV vaccination does not protect the newborn from getting the infection
□ RSV infection can cause serious disease for my newborn	□ RSV infection does not cause serious disease for my newborn
□ If the pediatrician would recommend it	□ I am concerned about the side effects of RSV vaccination
□ If RSV vaccination would be safe	□ If RSV vaccination would not be safe
□ If RSV vaccination would be effective	□ If RSV vaccination would be effective
□ I trust in vaccines	□ I do not trust in vaccines

Letter to participants

The Department of Experimental Medicine of the University of Campania 'Luigi Vanvitelli' and the Department of Health Sciences of the Magna Graecia University promoted a survey on attitudes towards future vaccination against the Respiratory Syncytial Virus among pregnant women. The questionnaire is anonymous. The information provided in the questionnaire cannot be traced back to the person who filled it in. Under Italian Legislative Decree 101/2018 on the protection of personal data, processing will only be carried out in aggregate form and will not involve any dissemination by name.

CONSENT FORM

Title of the study: "**Respiratory Syncytial Virus: willingness towards a future vac-cine among pregnant women in Italy**"

I, the undersigned

………………………………… ……………………….……… ……. / ……. / …….

Surname *Name* *date of birth*

And resident in

…………………………………………………..…………..…………………

Address

………………………………………. ………………………

City *Phone number*

declare that I

- voluntarily participate in the study, the purpose of which has been explained to me and of which I understand the purpose, the procedures to which I may be exposed, the possible risks and benefits, and the possible alternatives;
- have read the "Letter" forming part of this consent, which confirms what I have been told about the study;
- have had the opportunity to ask clarifying questions and have received satisfactory answers;
- have had ample time before deciding whether or not to participate;
- not have had any undue coercion in requesting my consent/not consenting by the investigator about my participation in the study;
- under Italian Legislative Decree no. 196 of 30.6.03 and GDPR no. 679/2016, I authorize the proposer of this study to process (in the sense specified by law) the personal and sensitive data concerning me, provided to the investigator, as necessary for my participation in the study in question.

Date ….. / …… / …..

Participant's sign ……………..
